# Astaxanthin Attenuates Homocysteine-Induced Cardiotoxicity *in Vitro* and *in Vivo* by Inhibiting Mitochondrial Dysfunction and Oxidative Damage

**DOI:** 10.3389/fphys.2017.01041

**Published:** 2017-12-12

**Authors:** Cun-dong Fan, Jing-yi Sun, Xiao-ting Fu, Ya-jun Hou, Yuan Li, Ming-feng Yang, Xiao-yan Fu, Bao-liang Sun

**Affiliations:** ^1^Key Lab of Cerebral Microcirculation in Universities of Shandong, Taishan Medical University, Taian, China; ^2^Wonju Severance Christian Hospital, Yonsei University Wonju College of Medicine, Wonju, South Korea; ^3^Department of Neurology, Affiliated Hospital of Taishan Medical University, Taian, China

**Keywords:** homocysteine, astaxanthin, cardiovascular diseases, mitochondrial dysfunction, oxidative damage

## Abstract

Homocysteine (Hcy) as an independent risk factor contributes to the occurrence and development of human cardiovascular diseases (CVD). Induction of oxidative stress and apoptosis was commonly accepted as the major mechanism in Hcy-induced cardiotoxicity. Astaxanthin (ATX) as one of the most powerful antioxidants exhibits novel cardioprotective potential against Hcy-induced endothelial dysfunction. However, the protective effect and mechanism of ATX against Hcy-induced cardiotoxicity in cardiomyocytes have not been elucidated yet. Herein, H9c2 rat cardiomyocytes and Hcy-injured animal model were employed in the present study. The MTT, flow cytometry analysis (FCM), TUNEL-DAPI and western blotting results all demonstrated that ATX significantly alleviated Hcy-induced cytotoxicity in H9c2 cells through inhibition of mitochondria-mediated apoptosis. The JC-1 and Mito-tracker staining both revealed that ATX pre-treatment blocked Hcy-induced mitochondrial dysfunction by regulating Bcl-2 family expression. Moreover, DCFH-DA and Mito-SOX staining showed that ATX effectively attenuated Hcy-induced oxidative damage via scavenging intracellular reactive oxygen species (ROS). Importantly, the ELISA and immunohistochemical results indicated that Hcy-induced cardiotoxicity *in vivo* was also significantly inhibited by ATX through inhibition of oxidative damage and apoptosis, and improvement of the angiogenesis. Taken together, our results demonstrated that ATX suppressed Hcy-induced cardiotoxicity *in vitro* and *in vivo* by inhibiting mitochondrial dysfunction and oxidative damage. Our findings validated the strategy of using ATX may be a highly efficient way to combat Hcy-mediated human CVD.

## Introduction

Cardiovascular diseases (CVD) as the leading causes of death globally represent one of the most challenges in clinic (Lee et al., 2016). Endothelial cells play key role in vascular homeostasis, and endothelial dysfunction contributed to the development of human CVD (Zhang et al., 2001; Ungvari et al., 2003; Austin et al., 2004). Homocysteine (Hcy) is an intermediate metabolite of cysteine and methionine. Large numbers of evidences have confirmed that elevated plasma levels of Hcy as an independent risk factor may induce endothelial dysfunction through oxidative stress and apoptosis, and eventually lead to the occurrence and development of human CVD (Almashhadany et al., 2015; Baggott and Tamura, 2015). However, Hcy-mediated toxicity toward cardiomyocytes was not well demonstrated, and the underlying mechanism remains elusive. Therefore, H9c2 rat cardiomyocytes and an Hcy-injured experimental animal model were employed to evaluate the potential cardiotoxicity and the underlying mechanism.

Astaxanthin (ATX) a red-orange carotenoid pigment represents one of the most powerful antioxidants and displays multiple biological activities, including anti-cancer, anti-inflammatory, anti-diabetic, immunomodulatory and neuroprotective activities (Hussein et al., 2006). Additionally, ATX also showed novel cardioprotective properities, and supplement of ATX in diet can decrease the risk of cardiovascular disease (Abdelzaher et al., 2016). Accumulated evidences have revealed that ATX had the potential to alleviate endothelial dysfunction through scavenging reactive oxygen species (ROS) and inhibiting oxidative damage (Sasaki et al., 2011; Zhao et al., 2011). Hence, ATX-mediated protection and mechanism in endothelial cells were well studied. But, little information about ATX-mediated protective potential in cardiomyocytes was available, and the protective mechanism was not well explored. Herein, the protective effects and mechanism of ATX against Hcy-induced cardiotoxicity in H9c2 rat cardiomyocytes and an experimental animal model were evaluated, and the results indicated that ATX attenuated Hcy-induced cardiotoxicity *in vitro* and *in vivo* by inhibiting mitochondrial dysfunction and oxidative damage, which validated its potential application in chemoprevention and chemotherapy of human CVD.

## Materials and methods

### Chemicals

DMEM-F12 medium, phosphate buffered solution (PBS) fetal bovine serum (FBS), DCFH-DA probe and mitochondria-targeted MitoSOX probe (M36008) were purchased from Invitrogen. Hcy, ATX, MTT, propidium iodide (PI) were obtained from Sigma. TUNEL-DAPI kit, Mito-SOX probe and BCA assay kit were purchased from Beyotime Institute of Biotechnology (Shanghai, China). Primary antibodies, including cleaved PARP (#94885), active-caspase-3 (#9664), active-caspase-7 (#8438), active-caspase-9 (#9507), Bax (#2772), Bad (#9292), Bcl-xL (#2764), Ser428-ATR (#2853), Ser15-p53 (#9284), total-p53 (#2524), and Ser139-histone (#9718) were all obtained from Cell Signaling Technology (Beverly, USA). Bcl-2 (#14-6992-82), CD-34 (#MA1-10202) and Ser-1981-ATM (#14-9046-82) were bought from Invitrogen (Carlsbad, USA). All solvents used were of high performance liquid chromatography (HPLC) grade.

### Cell culture and cell viability assay

H9c2 rat myocardial cells were obtained from ATCC company (USA). Cells were cultured with DMEM-F12 medium containing 10% FBS at 37° and 5% CO_2_ in a incubator. Cell viability with Hcy or/and ATX was detected by MTT assay. Briefly, H9c2 cells (8 × 10^3^ cells/well) seeded in 96-well plate were treated with Hcy (1–16 mM) for 72 h, or cells were treated with 8 mM Hcy for 6, 12, 24, 48, and 72 h. For protective treatment, cells were pre-treated with 0.5–8 μM ATX for 6 h and co-incubated with Hcy for 72 h. After treatment, 20 μl of MTT solution was added and incubated for another 5 h. Then the medium was removed and 150 μl of dimethyl sulfoxide (DMSO) was added. The cell viability was measured by detecting the absorbance at 570 nm. H9c2 cells morphology was observed by phase microscope. All data and images were obtained from three independent trials and were conducted in accordance with the relevant guidelines and regulations of Taishan Medical University.

### Flow cytometry analysis (FCM)

Cell apoptosis and cell cycle distribution in H9c2 cells were analyzed by FCM. Briefly, cells were pre-treated with or without 4 μM ATX for 6 and co-treated with 8 mM Hcy for 72 h. After treatment, cells were washed, collected and incubated with PI buffer at 37°C for 5 h in darkness. Then the stained cells were monitored by FCM. Cell cycle (G0/G1, S, and G2/M phases) was analyzed by Modfit software. The hypodiploid DNA content (Sub-G1 peak) was employed to quantify the apoptotic cell death. About 10^4^ cells/sample were recorded. All data and images were obtained from three independent trials and were conducted in accordance with the relevant guidelines and regulations of Taishan Medical University.

### TUNEL-DAPI staining

Cells apoptosis in H9c2 cells were also detected by TUNEL-DAPI staining. Briefly, Treated H9c2 cells seeded on a cover glass were fixed with 4% formaldehyde, and permeabilized with 0.1% Triton X-100. Then cells were incubated with TUNEL reaction solution for 1 h in darkness at 37°C. After incubation, cells were stained with 10 μg/ml DAPI for 10 min, and washed with PBS for three times. The cells apoptosis was detected by an inverted fluorescence microscope. The TUNEL-positive cells represent the apoptotic cells. All data and images were obtained from three independent trials and were conducted in accordance with the relevant guidelines and regulations of Taishan Medical University.

### Evaluation of mitochondrial dysfunction

Mitochondrial function was evaluated by the mitochondrial membrane potential (Δψ_m_) and mitochondrial morphology, which were examined by JC-1 and Mito-Tracker probes, respectively. Briefly, cells seeded in 6-cm plate were treated with ATX or/and Hcy. Cells after treatment were washed and incubated with 10 μM JC-1 or Mito-Tracker for 15 min in darkness. Then cells were washed and imaged under an inverted fluorescence microscope. The green fluorescence intensity after JC-1 staining was quantified by ImagePlus Software. All data and images were obtained from three independent trials and were conducted in accordance with the relevant guidelines and regulations of Taishan Medical University.

### Detection of ROS and superoxide anion

The intracellular ROS and superoxide anion were measured by DCFH-DA and Mito-SOX probes in live cells, respectively. Briefly, treated cells cultured on glass were washed and incubated with 10 μM DCFH-DA or 0.5 μM Mito-SOX. After reaction, cells were washed and observed by an inverted fluorescence microscope for detection of ROS (green fluorescence) and superoxide anion (red fluorescence). The images showed here were obtained from three independent experiments. All data and images were obtained from three independent trials and were conducted in accordance with the relevant guidelines and regulations of Taishan Medical University.

### Western blotting

Protein expression was examined by western blotting method. Briefly, cells after treatment were washed, collected and lysed by RIPA lysis buffer. The intracellular total protein was extracted by centrifugation at 11,000 *g* for 10 min at 4°C. Total protein after quantification was boiled and loaded (40 μg/lane) for separation by SDS-PAGE. Then protein was transferred onto a nitrocellulose membrane at 100 V for 1.5 h. Membrane subsequently was blocked, incubated with the primary antibody, and second antibody, respectively. Then the membrane was washed and the protein bands were visualized on an X-ray film using an enhanced ECL chemiluminescence system. The proteins expression was quantified by Quantity-One Software, and the proteins expression rate was labeled under the bands. All data and images were obtained from three independent trials and were conducted in accordance with the relevant guidelines and regulations of Taishan Medical University.

### *In vivo* study

The therapeutic effect of ATX against Hcy-induced cardiotoxicity was also evaluated *in vivo* in mouse. Briefly, 40 mice were divided into four groups (10 mice/group), and administrated with 5 mg/kg/day ATX or/and 300 mg/kg/day Hcy for 4 weeks. The control group was given equal normal saline. After administration, mouse was given euthanasia and the body weight and heart weight were measured. The contents of reduced glutathione (GSH-Rs) and malondialdehyde (MDA) in heart tissue were examined by ELISA kits according to the manufacture's instruction. Part of heart tissue was cut into 4-μM section for immunohistochemical assay (IHC). All the animal experiments were conducted in accordance with the relevant guidelines and regulations of Taishan Medical University.

### Statistical analysis

All data and images were done from three independent experiments at least. The statistical analysis was carried out by SPSS013.0 software. The significance between two groups was analyzed by two-tailed Student's test. The difference among three or more groups was analyzed by multiple comparisons. Bars with “^*^” or “^**^” represent the *P* < 0.05 or *P* < 0.01, respectively. Bars with different characters indicates the statistical different at *P* < 0.05 level.

## Results

### ATX alleviates Hcy-induced cytotoxicity in H9c2 cells

**Figure 1:**
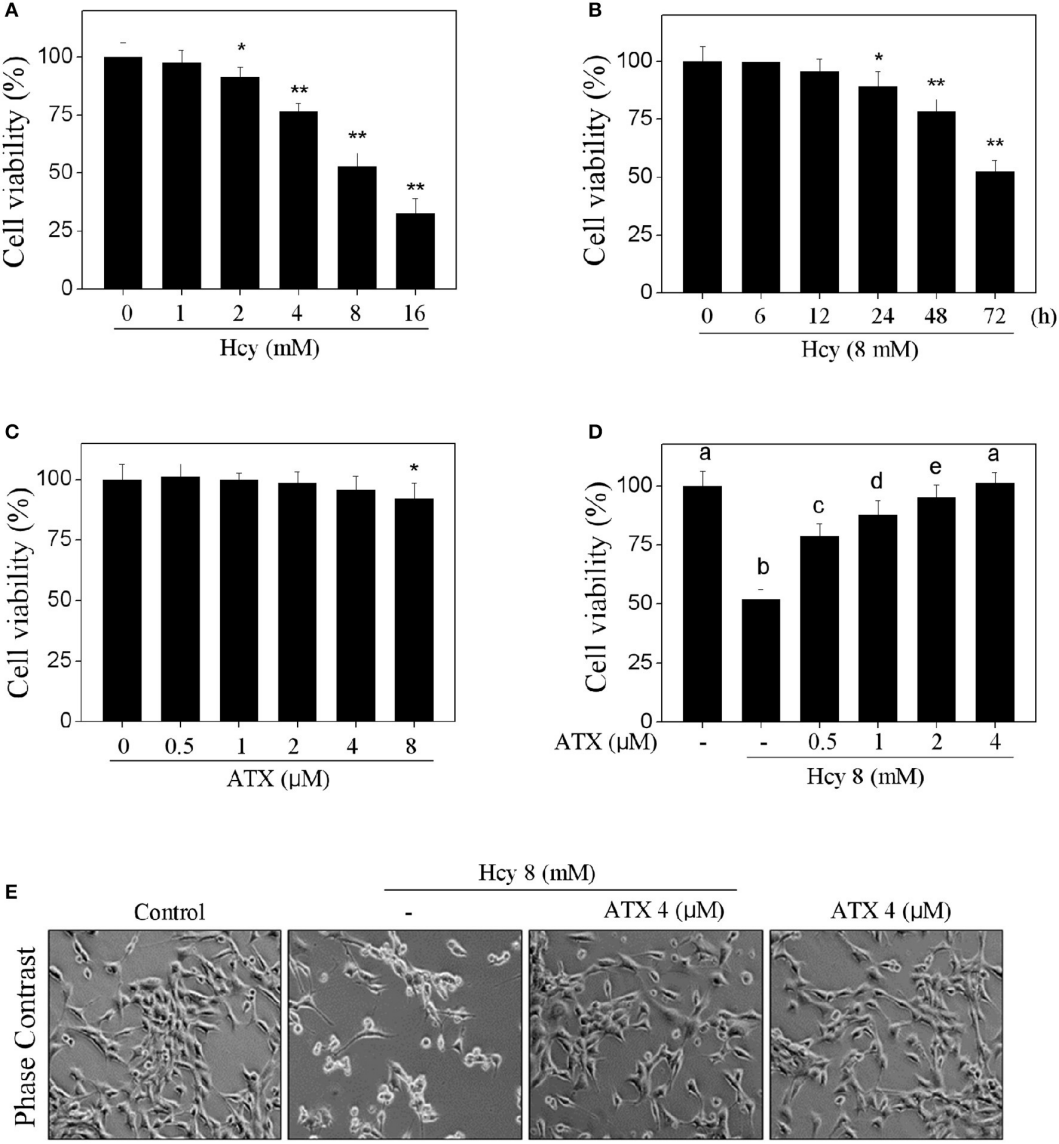
ATX alleviates Hcy-induced cytotoxicity in H9c2 cells. Dose-dependent **(A)** and time-dependent **(B)** cytotoxicity of Hcy toward H9c2 cells. H9c2 cells were treated with 0–16 mM Hcy for 72 h, or cells were exposed to 8 mM Hcy for 0–72 h. **(C)** Cytotoxicity of ATX toward H9c2 cells. Cells were treated with ATX (0–8 μM) for 72 h. **(D)** ATX alleviated Hcy-induced cytotoxicity in H9c2 cells. Cells were pre-treated with or without 0.5–4 μM ATX for 6 h and co-treated with 8 mM Hcy for 72 h. Cell viability after treatment was detected by MTT assay. **(E)** Morphological changes of H9c2 cells. Cells were treated with or without 4 μM ATX for 6 h, and co-incubated with 8 mM Hcy for 72 h. Cells after treatment were observed by phase contrast microscope (magnification, 200×). All data and images were obtained from three independent trials. Bars with “^*^” or “^**^” indicate the statistically different at the *P* < 0.05 and *P* < 0.01 level, respectively. Bars with different characters indicates the statistical different at *P* < 0.05 level.

The cytotoxicity of Hcy and possible protective effect of ATX on H9c2 cells were firstly examined by MTT assay. As shown in Figure 1 and Supplementary Figure 1, Hcy treatment alone significantly inhibited H9c2 cells viability in a dose- and time-dependent manner (Figures 1A,B). For instance, treatment of cells with 8 mM Hcy for 72 h significantly decreased the cell viability to 52.6%. However, pre-treatment of cells with ATX (1, 2 and 4 μM) for 6 h effectively blocked Hcy-induced cytotoxicity in H9c2 cells from 52.6% (Hcy) to 87.9, 95.4, and 99.8%, respectively (Figure 1D). ATX alone showed no obvious cytotoxicity toward H9c2 cells (Figure 1C). The improvement of cell morphology further confirmed this protective potential (Figure 1E). These results suggested that ATX has the potential to alleviate Hcy-induced cytotoxicity.

### ATX suppresses Hcy-induced apoptosis in H9c2 cells

**Figure 2:**
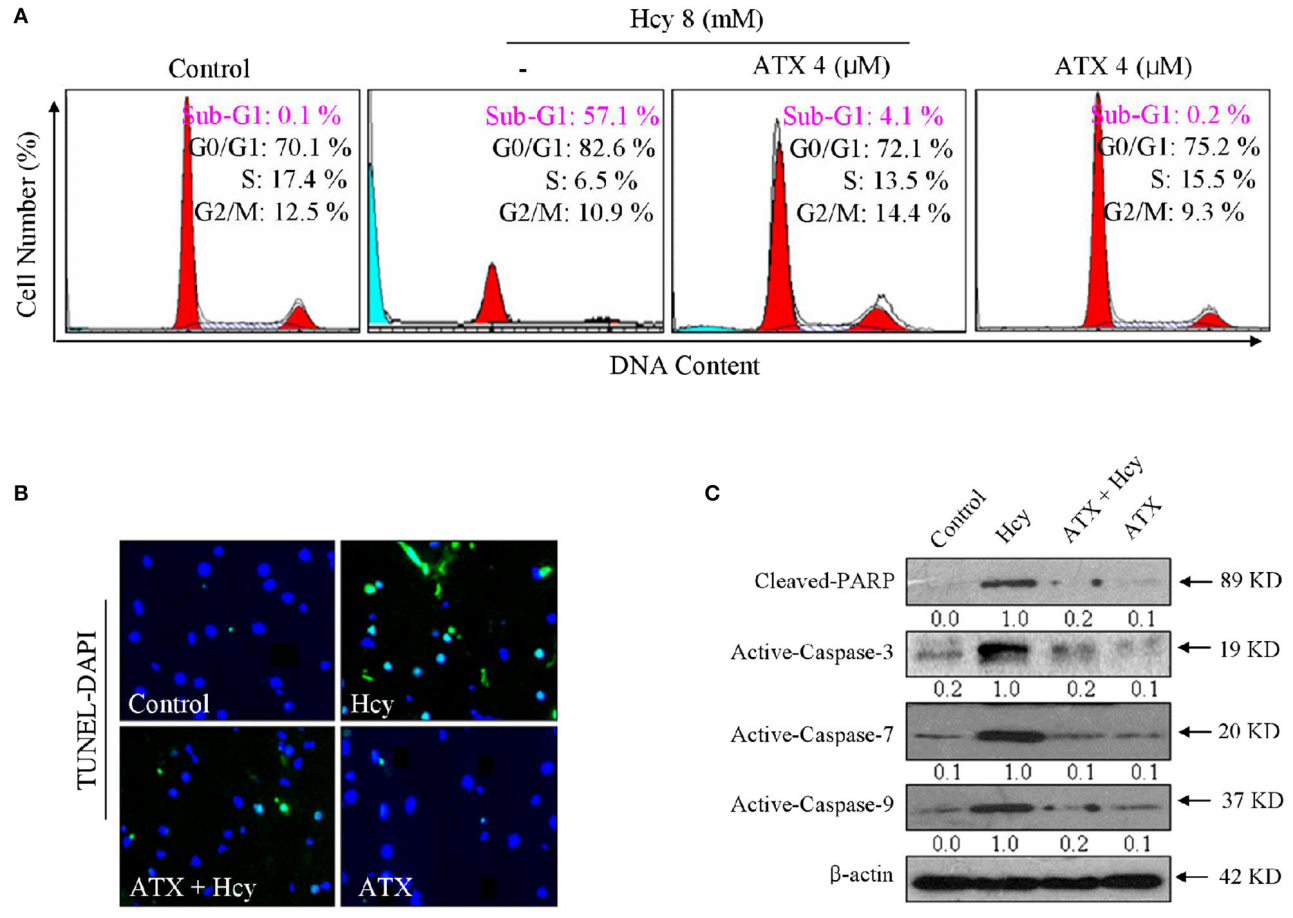
ATX suppresses Hcy-induced apoptosis in H9c2 cells. **(A)** FCM analysis of cell apoptosis and cell cycle distribution. H9c2 cells were treated with or without 4 μM ATX for 6 h, and co-incubated with 8 mM Hcy for 72 h. Cells after treatment were collected and fixed with 70% pre-cooled alcohol, and stained by PI solution and analyzed by FCM. The hypodiploid DNA content (Sub-G1 peak) was used to quantify the cell apoptosis. **(B)** TUNEL-DAPI staining. Cells after treatment were fixed with formaldehyde (4%) and administrated with the TUNEL staining kit as described in method section. The TUNEL-positive cells (green) indicated the apoptotic cells (magnification, 200×). **(C)** PARP and caspase expression. Total protein was prepared and the protein expression was examined by western blotting method. All data and images were obtained from three independent experiments.

We next evaluated Hcy-induced apoptosis and the possible protective mechanism of ATX on H9c2 cells by FCM. As shown in Figure 2A, Hcy alone caused apparent apoptosis, as reflected by the increase of Sub-G1 peak. However, the cell apoptosis in Hcy-treated cells was significantly attenuated by ATX pre-treatment from 52.6 to 4.1%. This protection of ATX on H9c2 cells was evaluated by TUNEL-DAPI co-staining. As shown in Figure 2B, Hcy treatment alone resulted in significant apoptosis, as convinced by the increase of TUNEL-positive cells. However, pre-treatment with ATX effectively prevented H9c2 cells from HCY-induced apoptosis. Moreover, the PARP cleavage and caspase activation were also conducted to investigate the underlying mechanism. As shown in Figure 2C, incubation of cells with Hcy alone induced dramatically poly-ADP-ribose polymerase (PARP) cleavage and the activation of caspase-3, -7, and -9. The activation of caspase-7 and caspase-9 indicated that Hcy activated the mitochondria-mediated apoptosis. The slight activation of capase-8 indicated that Hcy also triggered death receptor-mediated apoptosis, but not dominant (Supplementary Figure 2). As expected, ATX pre-treatment markedly inhibited Hcy-induced PARP cleavage and caspase activation. Taken together, these results above indicated that ATX suppressed the mitochondria-mediated apoptosis in Hcy-treated H9c2 cells.

### ATX blocks Hcy-induced mitochondrial dysfunction by balancing Bcl-2 family

**Figure 3:**
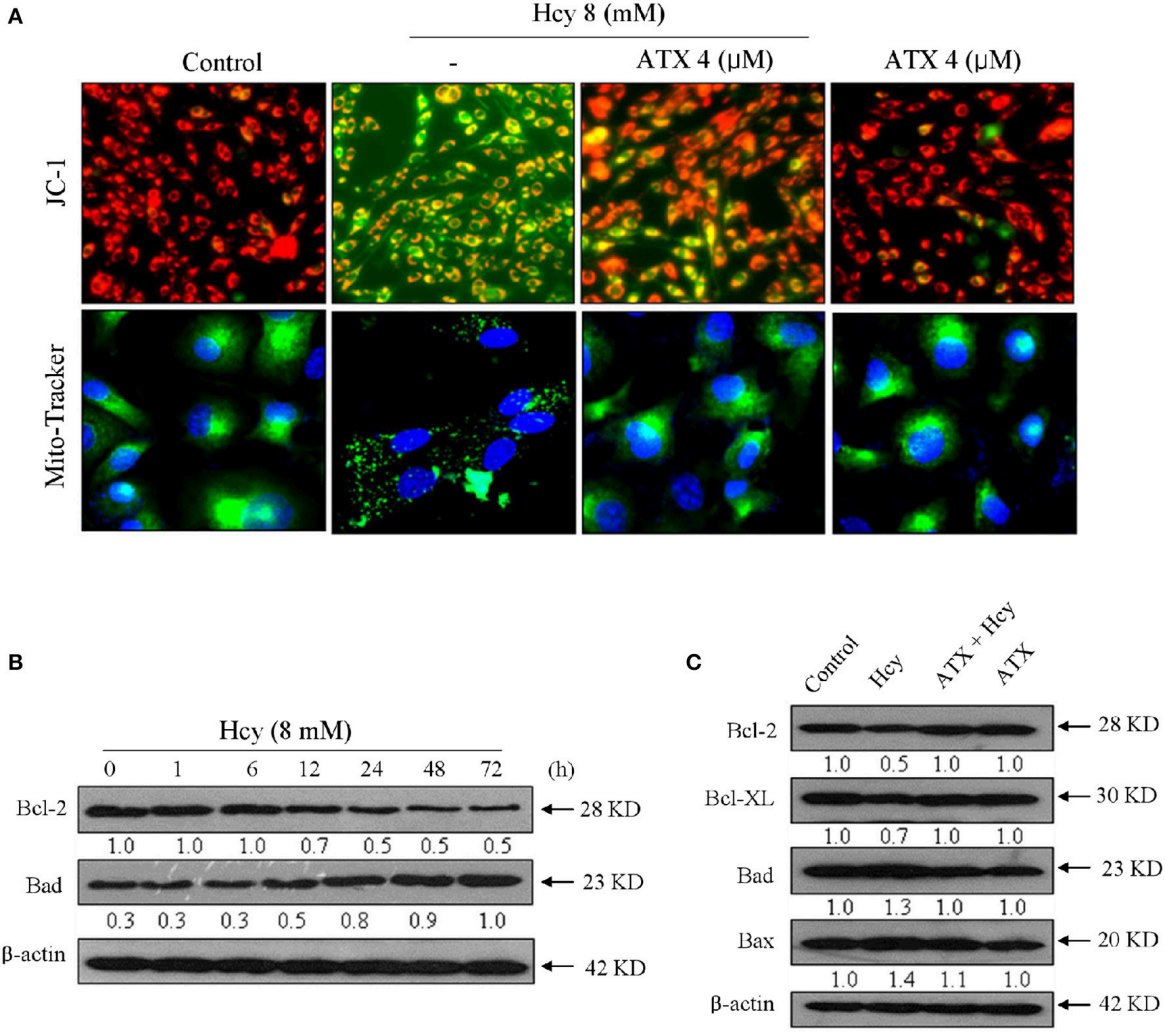
ATX blocks Hcy-induced mitochondrial dysfunction by regulating Bcl-2 family. **(A)** ATX blocked Hcy-induced the depletion of Δψ_m_ and mitochondrial fragmentation. The Δψ_m_ and mitochondrial morphology were detected by JC-1 and Mito-Tracker probes, respectively. The experiment details were conducted according to the method section. **(B)** Time-dependent inhibition of Hcy against Bcl-2 and Bad expression. Cells were treated with 8 mM Hcy for indicated time. **(C)** Protective effects of ATX on Bcl-2 family members in Hcy-treated cells. Protein expression was examined by western blotting method. All images were obtained from three independent trials.

To elucidate the role of mitochondria in Hcy-induced apoptosis of H9c2 cells, the Δψ_m_ and mitochondrial morphology were detected by JC-1 and Mito-tracker probes, respectively. As shown in Figure 3A, Hcy treatment induced significant loss of Δψ_m_, as reflected by the fluorescent shift from red to green. ATX pre-treatment completely improved the Δψ_m_ in Hcy-treated cells. The statistical result further confirmed this protective effect (Supplementary Figure 3). Moreover, Hcy treatment also caused obvious mitochondrial fragmentation, as demonstrated by the mitochondrial morphological changes from protonema to punctiform. Interestingly, ATX pre-treatment completely blocked Hcy-induced mitochondrial fragmentation.

Bcl-2 family plays important role in regulating mitochondrial permeability and inducing apoptosis. Therefore, a time-course effect of Hcy on Bcl-2 family members was investigated. As show in Figure 3B, Hcy treatment caused continuous down-regulation of Bcl-2 at 12 h. Bad expression in Hcy-treated H9c2 cells showed significant up-regulation at 12 h. However, the expression level of pro-apoptosis (Bax and Bad) and pro-survival (Blc-2 and Bcl-XL) in Hcy-treated cells were effectively normalized by ATX pre-treatment (Figure 3C). Taken together, these results revealed that ATX blocked Hcy-induced mitochondrial dysfunction by regulating Bcl-2 family proteins.

### ATX attenuates Hcy-induced oxidative damage in H9c2 cells

**Figure 4:**
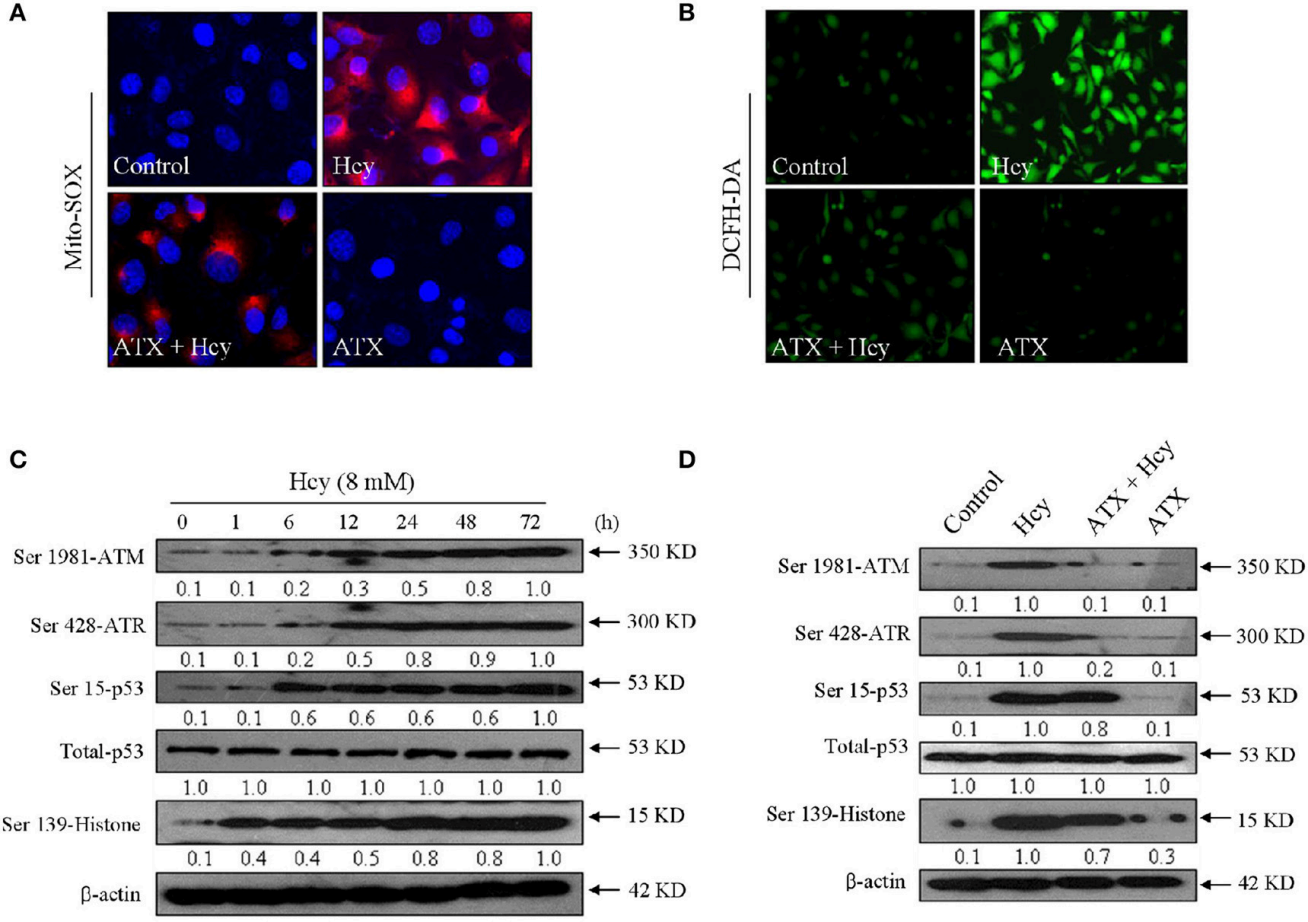
ATX attenuates Hcy-induced oxidative damage in H9c2 cells. ATX inhibited intracellular superoxide anion **(A)** and ROS **(B)** generation. The intracellular superoxide anion and ROS were measured by Mito-SOX and DCFH-DA probes, respectively. The experiment details were conducted according to the method section. **(C)** Time-dependent activation of Hcy on DNA damage. **(D)** ATX attenuated Hcy-induced DNA damage. Protein expression was examined by western blotting method. All images were obtained from three independent trials.

Accumulated evidences have confirmed that Hcy could trigger oxidative damage through inducing ROS accumulation. Therefore, the oxidative status in Hcy-treated H9c2 cells was investigated. Primarily, the intracellular superoxide anion and ROS were detected by Mito-SOX (a mitochondria-targeted red probe) and DCFH-DA (green), respectively. As shown in Figures 4A,B, Hcy treatment caused significant accumulation of superoxide anion and ROS, as demonstrated by the enhanced red fluorescence and green fluorescence in H9c2 cells, respectively. Secondly, Hcy-induced DNA damage was also detected. As shown in Figure 4C, cells treated with 8 mM Hcy showed significant phosphorylation activation of ataxia telangiectasia mutated (ATM) (Ser1981), and Rad3-related (ATR) (Ser428), p53 (Ser15), and histone (Ser139) in a time-dependent manner, indicating that Hcy induced oxidative damage in H9c2 cells. However, pre-treatment with ATX inhibited Hcy-induced generation of ROS and superoxide anion. The statistical results further confirmed ATX's anti-oxidative effect (Supplementary Figure 4). ROS inhibition by ATX eventually attenuated Hcy-induced oxidative damage (Figure 4D). Taken together, these results above demonstrated that ATX attenuated Hcy-induced oxidative damage through inhibition ROS overproduction.

### ATX inhibits Hcys-induced cardiotoxicity *in vivo*

**Figure 5:**
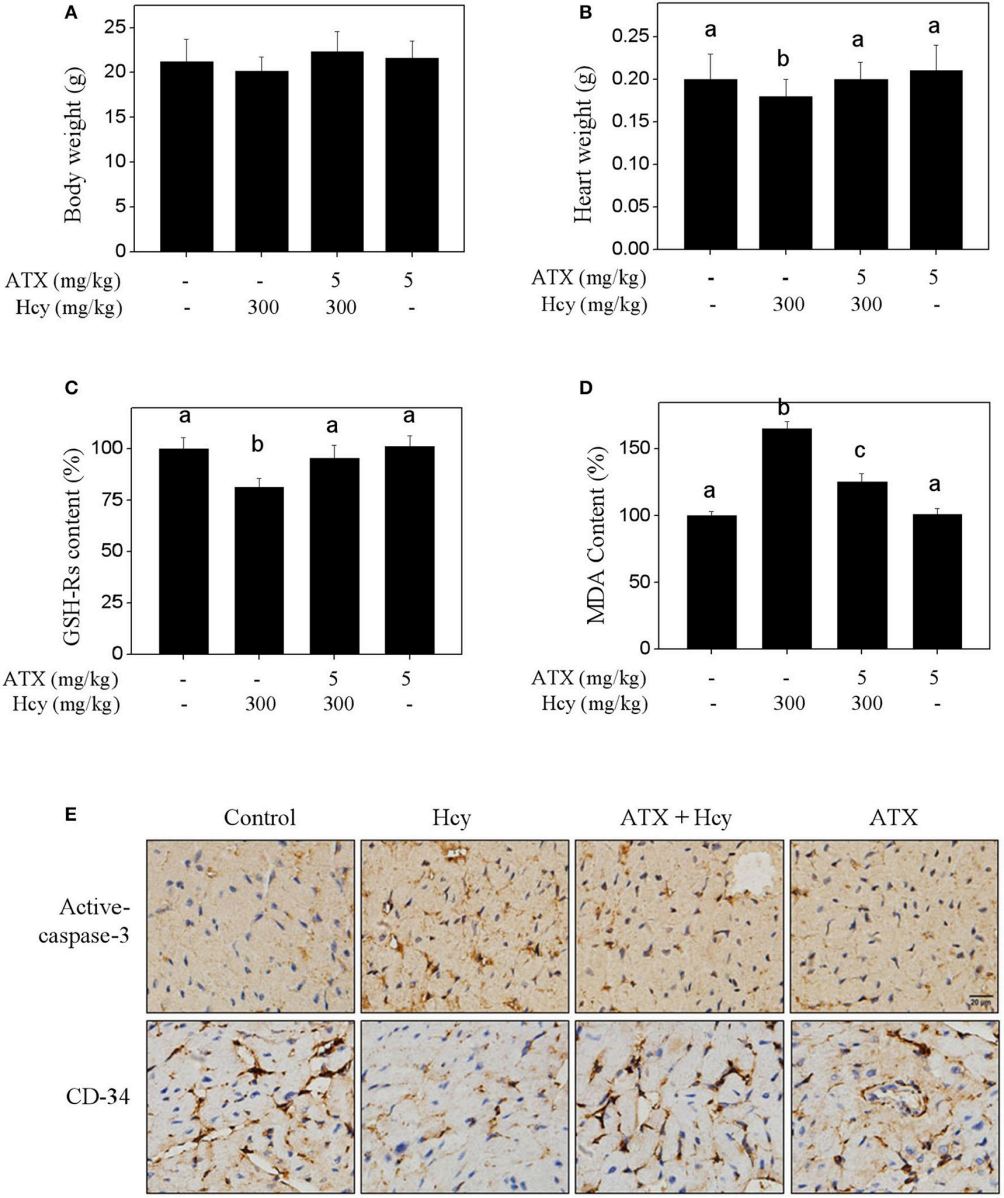
ATX inhibits Hcy-induced cardiotoxicity *in vivo*. Effects of ATX or/and Hcy on the mice body weight **(A)** and heart weight **(B)** *in vivo*. Mice were given ATX (5 mg/kg/day) or/and Hcy (300 mg/kg/day) for 2 weeks. After administration, the mice body weight and heart weight were both examined. Effects of ATX or/and Hcy on the content of GSH-Rs **(C)** and MDA **(D)**. Total protein was extracted from mice heart tissue, and the content of GSH-Rs and MDA were examined by ELISA kits. **(E)** IHC assay of apoptosis and angiogenesis *in vivo*. Heart tissue were separated and cut into 4-μm section. The expressions of active-caspase-3 and CD-34 in heart tissue were detected by IHC method. All data and images were obtained from three independent trials. Bars with different characters indicates the statistical different at *P* < 0.05 level.

To validate the *in vivo* protective effect and possible mechanism of ATX against Hcy-induced cardiotoxicity, mouse administrated with ATX or/and Hcy were employed. The results showed that Hcy treatment *in vivo* caused slight decrease in mice heart weight, but not affect the mice body weight (Figures 5A,B). Hcy treatment also decreased the content of reduced-glutathione (GSH-Rs) (Figure 5C) and increased the content of MDA (Figure 5D), indicating the induction of oxidative damage by Hcy. IHC analysis revealed that Hcy treatment *in vivo* induced significant myocardial apoptosis (active-caspase-3 staining) and inhibited angiogenesis (CD-34 staining). Importantly, ATX treatment *in vivo* effectively inhibited the oxidative damage and myocardial apoptosis, and improved the angiogenesis in Hcy-treated mouse (Figure 5E), which consisted with the *in vitro* mechanism. These results suggested that ATX inhibited Hcy-induced cardiotoxicity *in vivo*.

## Discussion

Human CVD represents the leading causes of death globally, and therapy of human CVD has always remains one of the biggest challenges in clinic. High levels of plasma Hcy as an independent risk factor contributes to the occurrence and development of CVD (Feng and Xu, 2017; Markišić et al., 2017; Rudreshkumar et al., 2017). Accumulated studies have indicated that Hcy as an intermediate metabolite of cysteine and methionine can cause endothelial dysfunction (Zhang et al., 2017). For instance, patients with hyperhomocysteinemia usually show decreased numbers of endothelial cells with impaired endothelial activities in endothelial proliferation, migration and adhesion, which all do harm to human heart health (Jamaluddin et al., 2007; Almashhadany et al., 2015). Experimentally, Hcy has been proved to be associated with disturbed cardiac substrate metabolism, mitochondrial dysfunction and adverse cardiac remodeling with increased myocardial stiffness (Joseph et al., 2003; Devi et al., 2006; Suematsu et al., 2007). However, whether Hcy may show similar toxic effect on cardiomyocytes is unknown. The underlying mechanism has not been well elucidated yet. Hence, in the present study, Hcy-induced cardiotoxicity and underlying mechanism were evaluated in H9c2 rat cardiomyocytes and Hcy-injured animal model, which are both accepted as good models for exploring the cardiotoxicity *in vitro* and *in vivo*. The results indicated that Hcy displayed significant cardiotoxicity *in vitro* and *in vivo*, as reflected by the cytotoxicity and cells apoptosis of H9c2 cells *in vitro*, and inhibition of angiogenesis *in vivo*. Meanwhile, we revealed the toxic mechanism that Hcy-induced cardiotoxicity was mainly achieved by triggering mitochondrial dysfunction and oxidative damage.

ROS, including hydroxyl radical, hydrogen peroxide, and superoxide anion (Sun et al., 2013), all play important roles in mediating cell signaling and maintaining cell homeostasis (Fan et al., 2014). The balance of anti-antioxidant and pro-antioxidant system ultimately decides the intracellular ROS level (Li et al., 2015; Wang et al., 2016a,b; Fan et al., 2017a). Oxidative stress was accepted as a key index in the pathology of human CVD (Castardo-de-Paula et al., 2017; Ghosh et al., 2017; Paul et al., 2017; Ramirez-Lee et al., 2017). Inhibition of oxidative damage represents of one of the most efficient strategies in treatment of human CVD. Therefore, searching novel agents with potential antioxidant activity and low side effects to combat human CVD is urgently needed.

ATX exhibits excellent pharmacological properties, including anti-cancer, anti-inflammatory, anti-diabetic, immunomodulatory and neuroprotective activities, which are all based on its antioxidant activity (Hussein et al., 2006; Abdelzaher et al., 2016). Large number of evidences have supported that ATX had the potential in chemoprevention and chemotherapy of several human diseases through eliminating ROS and attenuating oxidative damage in all kinds of cell and animal models (Wu et al., 2015). However, ATX-mediated protective effects against Hcy-induced cardiotoxicity have not been reported, and the underlying mechanism remains unclear.

The underlying mechanism has not been well elucidated yet. Hence, in the present study, Hcy-induced cardiotoxicity and underlying mechanism were evaluated in H9c2 rat cardiomyocytes and Hcy-injured animal model. In the present study, Hcy treatment caused significantly ROS over-production and eventually triggered oxidative damage *in vitro* and *in vivo*. However, ATX as a powerful inhibitor of ROS effectively inhibited ROS accumulation and blocked Hcy-induced oxidative damage *in vitro* and *in vivo*, as convinced by the decreased level of phosphorylation activation of ATM (Ser1981), ATR (Ser428), p53 (Ser15), and histone (Ser139). These results indicated that ROS as early apoptotic event was involved in Hcy-induced H9c2 cells apoptosis, and ATX can act as ROS inhibitor to suppress Hcy-induced apoptosis and oxidative damage.

Mitochondria integrates the intrinsic and extrinsic signals and plays important role in lunching mitochondria-mediated apoptosis (Zhu et al., 2016). Mitochondrial membrane permeabilization acts as an important event in inducing cell apoptotic death in response to apoptotic stimuli (Kroemer et al., 2007; Zhu et al., 2016; Fan et al., 2017b). Bcl-2 family, including the pro-apoptotic and anti-apoptotic members, has been confirmed as essential factors in regulating mitochondria-mediated apoptosis pathway (Cory and Adams, 2002). Increasing studies supported that loss of Δψ_m_ was highly multiple associated with the capsases activation and Bcl-2 family expression (Kroemer et al., 2007). For instance, Bcl-2 and Bcl-XL can bind to the out membrane of mitochondria of healthy cells (Cory and Adams, 2002). Bax can form homo-oligomers with Bak to permeabilize the out membrane of mitochondria and cause the depletion of Δψ_m_ (Wei et al., 2001; Cory and Adams, 2002). In the present study, our result suggested that Hcy treatment caused significant mitochondria-mediated apoptosis with involvement of mitochondrial dysfunction. Immunofluorescent staining of mitochondria affirmed that the loss of Δψ_m_ and mitochondrial morphological changes both contributed to the mitochondrial dysfunction in Hcy-treated H9c2 cells. The western blotting results revealed that Hcy-induced mitochondrial dysfunction was associated with the imbalance of Bcl-2 family expression. However, ATX pre-treatment prevented H9c2 cells from Hcy-induced mitochondrial dysfunction and the imbalance of Bcl-2 family expression, and eventually reversed Hcy-induced apoptosis. Therefore, we concluded that ATX blocked Hcy-induced mitochondria-mediated apoptosis by stabilizing Bcl-2 family expression.

In summary, the present study demonstrated that ATX suppressed Hcy-induced cardiotoxicity *in vitro* and *in vivo* by inhibiting mitochondrial dysfunction and oxidative damage. Our findings validated the potential therapeutic role of ATX in chemoprevention and chemotherapy of Hcy-mediated human CVD.

## Ethical statement

The study entitled “Astaxanthin Attenuates Homocysteine-Induced Cardiotoxicity *in Vitro* and *in Vivo* by Inhibiting Mitochondrial Dysfunction and Oxidative Damage” was performed in Key Lab of Cerebral Microcirculation in Universities of Shandong, Taishan Medical University. All the *in vitro* and *in vivo* experiments were performed in accordance with the relevant guidelines and regulations of Taishan Medical University. Especially, the *in vivo* experiments were approved by the Taishan Medical University Ethics Committee. All surgery was performed under 10% chloral hydrate, and every effort was made to minimize suffering.

## Author contributions

C-dF and B-lS designed the experiments. J-yS, X-tF, Y-jH, and YL performed the *in vitro* and *in vivo* experiments. M-fY and X-yF analyzed the data and images. C-dF and X-yF wrote the manuscript. All authors reviewed the manuscript.

### Conflict of interest statement

The authors declare that the research was conducted in the absence of any commercial or financial relationships that could be construed as a potential conflict of interest.
