# Supplementary material for: Astaxanthin Attenuates Homocysteine-Induced Cardiotoxicity in Vitro and in Vivo by Inhibiting Mitochondrial Dysfunction and Oxidative Damage
Source: Front Physiol. 2017 Dec 12;8:1041. doi: 10.3389/fphys.2017.01041 (PMC5733103; doi:10.3389/fphys.2017.01041)
Supplement: Supplementary file 1 [file DataSheet1.DOC]

Supporting Information for

**Astaxanthin Attenuates Homocysteine-Induced Cardiotoxicity *In Vitro* and *In Vivo* by Inhibiting Mitochondrial Dysfunction and Oxidative Damage**

Cun-dong Fan^1‡^, Jing-yi Sun^2‡^, Xiao-ting Fu^1‡^, Ya-jun Hou^1^, Yuan Li^1^, Ming-feng Yang^1^, Xiao-yan Fu^1*^, Bao-liang Sun^1,3*^,

^1^ Key Lab of Cerebral Microcirculation in Universities of Shandong, Taishan Medical University, Taian, Shandong, 271000, China

^2^ Wonju Severance Christian Hospital, Yonsei University Wonju College of Medicine, Wonju, Gangwon 220-701, Korea

^3^ Department of Neurology, Affiliated Hospital of Taishan Medical University, Taian 271000, Shandong, China

**‡** These authors contributed equally to this work.

***Corresponding authors.**

**Xiao-yan Fu**, Yingsheng East Road 2, Taishan Medical University, Taian, Shandong, 271000, China. Email: txyfu66@163.com, Tel: +86-538-6230027, Fax: +86-538-6230027

**Bao-liang Sun**, Yingsheng East Road 2, Taishan Medical University, Taian, Shandong, 271000, China. Email: [tblsun66@163.com](mailto:tblsun66@163.com), Tel: +86-538-6230030, Fax: +86-538-6230030

**Supplementary Figure 1. Cell morphology of H9c2 cells after treatment of Hcy.** H9c2 cells were treated with 2, 4 and 8 mM Hcy for 72 h. The cell morphology was observed by phase microscope. All images were obtained from three independent trials.

**Supplementary Figure 2. ATX attenuates Hcy-induced caspase activation.** H9c2 cells were treated with or without 4 μM ATX for 6 h, and co-incubated with 8 mM Hcy for 72 h. Then, total protein was extracted and the activation of caspase-9 and caspase-8 was detected by Ac-LEHD-AMC and Ac-IETD-AMC, respectively. All data were expressed as mean ± S.D. Bars with “**” indicates statistically different at the *P*<0.05 level.

**Supplementary Figure 3. ATX completely blocked Hcy-induced depletion of ∆ψ_m_.** H9c2 cells were treated with or without 4 μM ATX for 6 h, and co-incubated with 8 mM Hcy for 72 h. The loss of mitochondrial membrane potential (∆ψ_m_) was detected by measuring the increase of green fluorescence. The green fluorescence intensity was analyzed by Image-Plus software. All data were expressed as mean ± S.D. Bars in the figures with different characters are statistically different at the *P*<0.05 level.

**Supplementary Figure 4. ATX inhibted Hcy-induced accumulation of superoxide anion (A) and ROS (B).** H9c2 cells were treated with or without 4 μM ATX for 6 h, and co-incubated with 8 mM Hcy for 72 h. The generation of superoxide anion and ROS was detected by measuring the red and green fluorescence using Mito-SOX and DCFH-DA probes, respectively. The fluorescence intensity was analyzed by Image-Plus software. All data were expressed as mean ± S.D. Bars in the figures with different characters are statistically different at the *P*<0.05 level.
